# Gut Microbiota Modulates the Protective Role of Ginsenoside Compound K Against Sodium Valproate-Induced Hepatotoxicity in Rat

**DOI:** 10.3389/fmicb.2022.936585

**Published:** 2022-07-07

**Authors:** Luping Zhou, Xiangchang Zeng, Jianwei Liao, Lulu Chen, Dongsheng Ouyang

**Affiliations:** ^1^Department of Clinical Pharmacology, Xiangya Hospital, Central South University, Changsha, China; ^2^Hunan Key Laboratory of Pharmacogenetics, Institute of Clinical Pharmacology, Central South University, Changsha, China; ^3^Engineering Research Center of Applied Technology of Pharmacogenomics, Ministry of Education, Changsha, China; ^4^National Clinical Research Center for Geriatric Disorders, Changsha, China; ^5^The Cancer Hospital of the University of Chinese Academy of Sciences (Zhejiang Cancer Hospital), Institute of Basic Medicine and Cancer (IBMC), Chinese Academy of Sciences, Hangzhou, China; ^6^Hunan Key Laboratory for Bioanalysis of Complex Matrix Samples, Changsha, China

**Keywords:** sodium valproate, ginsenoside compound K, hepatotoxicity, gut microbiota, *Akkermansia muciniphila*

## Abstract

This study aimed to investigate the potential role of gut microbiota in the hepatotoxicity of sodium valproate (SVP) and the protective effect of ginsenoside compound K (G-CK) administration against SVP-induced hepatotoxicity in rats. Measurements of 16S rRNA showed that SVP supplementation led to a 140.749- and 248.900-fold increase in the relative abundance of *Akkermansia muciniphila* (*A. muciniphila*) and *Bifidobacterium pseudolongum* (*B. pseudolongum*), respectively (*p* < 0.05). The increase in *A. muciniphila* was almost completely reversed by G-CK treatment. The relative abundance of *A. muciniphila* was strongly positively correlated with aspartate transaminase (AST) and alanine aminotransferase (ALT) levels (*r* > 0.78, *p* < 0.05). The PICRUSt analysis showed that G-CK could inhibit the changes of seven pathways caused by SVP, of which four pathways, including the fatty acid biosynthesis, lipid biosynthesis, glycolysis/gluconeogenesis, and pyruvate metabolism, were found to be negatively correlated with AST and ALT levels (*r* ≥ 0.70, *p* < 0.01 or < 0.05). In addition, the glycolysis/gluconeogenesis and pyruvate metabolism were negatively correlated with the relative abundance of *A. muciniphila* (*r* > 0.65, *p* < 0.01 or < 0.05). This alteration of the gut microbiota composition that resulted in observed changes to the glycolysis/gluconeogenesis and pyruvate metabolism may be involved in both the hepatotoxicity of SVP and the protective effect of G-CK administration against SVP-induced hepatotoxicity. Our study provides new evidence linking the gut microbiota with SVP-induced hepatotoxicity.

## Introduction

Valproic acid and its derivatives function as effective anticonvulsants. They are also used for the treatment of several other conditions, including bipolar disorder, migraines, neuropathic pain, various forms of headache, Alzheimer's disease, multiple sclerosis, viral infections, and a wide variety of cancers (De Souza and Chatterji, 2015; Mishra et al., 2021; Stakisaitis et al., 2022). However, the potential adverse effects associated with valproic acid and its derivatives have been reported, either as monotherapy or polytherapy with other antiepileptic drugs or antipsychotic drugs (Nanau and Neuman, 2013; Hakami, 2021). The hepatotoxicity of valproic acid and its derivatives is a fatal adverse drug reaction identified as the third most common cause of drug-induced liver fatalities by the World Health Organization (Vidaurre et al., 2017). To find solutions to this outcome from the usage of such drugs, the mechanisms and effective interventions for hepatotoxicity of valproic acid and its derivatives have been a long-term focus for many researchers.

Ginsenoside compound K [20-O-beta-D-glucopyranosyl-20(S)-protopanaxadiol; also known by the names M1, compound K, IH901, CK, and G-CK] is a main active metabolite of Panax notoginseng saponins (Kim, 2013). It possesses favorable drug properties according to preclinical and phase I clinical trials (Registration number: ChiCTR-TRC-14004824 and ChiCTR-IPR-15006107) (Chen et al., 2017a,b) and multiple pharmacological activities. These include anticarcinogenic, antiangiogenic, antidiabetic, anti-inflammatory, anti-allergic, antiaging, and neuroprotective and hepatoprotective activities (Yang et al., 2015; Liu et al., 2022). Our previous research has shown that G-CK could alleviate the hepatotoxicity of sodium valproate (SVP) (Zhou et al., 2020a, 2021), while the underlying mechanism is not yet fully understood.

The gastrointestinal tract of a mammal contains a vast, complex, and dynamic consortium of microorganisms known collectively as the gut microbiota, also known as the ‘second genome’ of the human body. It is widely recognized to play an important role in coordinating the healthy and diseased states of the host. Recently, the composition of gut microbiota has been linked to the hepatotoxicity of atrazine (Liu et al., 2021), 1,4-dioxane (Zhou et al., 2020b), difenoconazole (Jiang et al., 2020), geniposide (Li et al., 2019), palmitic acid (Ding et al., 2018), acetaminophen (Gong et al., 2018), tacrine (Yip et al., 2018), N, N-dimethylformamide (Zhang et al., 2017), and PCB126 (Chi et al., 2019). In addition, the gut microbiota has also been reported to mediate the hepatoprotective effects of many forms of interventions (Meng et al., 2018; Yang et al., 2021). The role of gut microbiota presents a new research direction for exploring the mechanisms and effective interventions of drug-induced liver injury.

At present, the relationship between the hepatotoxicity of valproic acid or its derivatives and gut microbiota remains unclear, but valproic acid or SVP has been shown to cause a change in the composition of gut microbiota in rats and enhance or reduce the production of some fatty acids in microorganisms (Cussotto et al., 2019; Poolchanuan et al., 2020). The valproic acid rat model of autism can mimic the microbiome features of autism (Liu et al., 2018). Numerous studies have focused on the role of gut microbiota in the metabolism of ginsenosides to G-CK; however, less is known about the effects of G-CK on gut microbiota. Shao et al. recently reported that the gut microbiota contributes to the anti-colorectal cancer effect of G-CK (Shao et al., 2022). These findings led us to speculate that the hepatotoxicity of valproic acid or its derivatives and the hepatoprotective effect of G-CK may be related to gut microbiota.

Based on the above facts, this study aimed to investigate the effects of SVP either alone or combined with G-CK on gut microbiota composition, the relationship between the changes in gut microbiota composition and SVP-induced hepatotoxicity, and the protective effect of G-CK administration against SVP-induced hepatotoxicity.

## Materials and Methods

### Drugs and Chemicals

Ginsenoside compound K with 98% purity was provided by Hisun Pharmaceutical Co., Ltd. (Taizhou, China) and was prepared as a suspension in 0.5% sodium carboxymethylcellulose (CMC-Na) for intragastric administration. SVP was obtained from Hunan Xiangzhong Pharmaceutical Co., Ltd. (Shaoyang, China) and was dissolved in physiological saline for intragastric administration. The Stool DNA Isolation Kit was purchased from Qiagen (Shanghai, China).

### Animal Experiment

The fecal samples and liver injury-related indices including plasma aspartate transaminase (AST), alanine aminotransferase (ALT), and alkaline phosphatase (ALP) levels were obtained from the control (Con), 500 mg/kg SVP twice daily, and combined 320 mg/kg G-CK once daily, and SVP (G-CK + SVP) groups of a previous study (Zhou et al., 2020a). The animal study was reviewed and approved by the Laboratory Animal Ethics Committee of Servicebio in strict accordance with the National Institute of Health Guide for the Care and Use of Laboratory Animals (NIH Publications NO. 80-23, revised 1996).

### Gut Microbiota Analysis

Total bacterial genomic DNA was extracted from the fecal samples using a stool DNA Isolation Kit, following the manufacturer's instructions. Extracts were then treated with DNase-free RNase to eliminate RNA contaminants. Both the DNA yield and quality were measured by using a NanoDrop spectrophotometer, and the DNA integrity was analyzed by 1% agarose gel electrophoresis. The extracted DNA was amplified using universal primers (515F 5′-GTGYCAGCMGCCGCGGTAA-3′ and 806R 5′-GGACTACNVGGGTWTCTAAT-3′) to obtain the V4 regions of the 16S rRNA gene (Apprill et al., 2015; Parada et al., 2016). The reaction mixtures were set up using 25 μl of PCR Master Mix, 4 μl of each primer (10 μM), 30 ng of template DNA, and added with water up to 50 μl. The reaction parameters were as follows: 98°C pre-denaturation for 3 min, 95°C denaturation for 30 s, 55°C annealing for 45 s, 72°C extension for 45 s, for 40 cycles, followed by a final amplification step of 7 min at 72°C. High-throughput sequencing of amplicons was performed by the BGI technology company (Shenzhen, China) using the Illumina HiSeq 2500 system. Alpha diversity (within sample diversity) and beta diversity (whole community composition/inter-sample diversity) estimates were calculated by using Mothur (version 1.31.2). Alpha diversity indices used were as follows: Chao1, ACE, and Shannon indices. The Euclidean distance and Bray–Curtis matrix at the OTU level were used to explore the beta diversity. Principal components analysis (PCA) and principal coordinate analysis (PCoA) were further used to visualize the beta diversity. The LEfSe analysis was used to screen potential bacterial biomarkers related to the protective effect of G-CK against SVP-induced liver injury. The function of gut microbiota was predicted through the use of the PICRUSt software package. The data presented in the study are deposited in the NCBI repository, accession number PRJNA837380.

### Statistical Analysis

Statistical analysis was performed using GraphPad Prism (version 9.0.0) and R studio (version 3.1.1). Data were expressed as means ± SD, except for Chao1, ACE, and Shannon, which were presented as medians and min-to-max values. Alpha diversity was analyzed with Wilcoxon rank-sum tests. Kruskal–Wallis non-parametric test with Benjamini–Hochberg's correction was applied for the statistical analysis of the relative abundance of gut microbiota between groups. Spearman's correlation was carried out to further screen the relationship between gut microbiota and liver injury-related indices. Data with *p* < 0.05 were considered to have statistical differences.

## Results

### Gut Microbiota Composition

**Figure 1:**
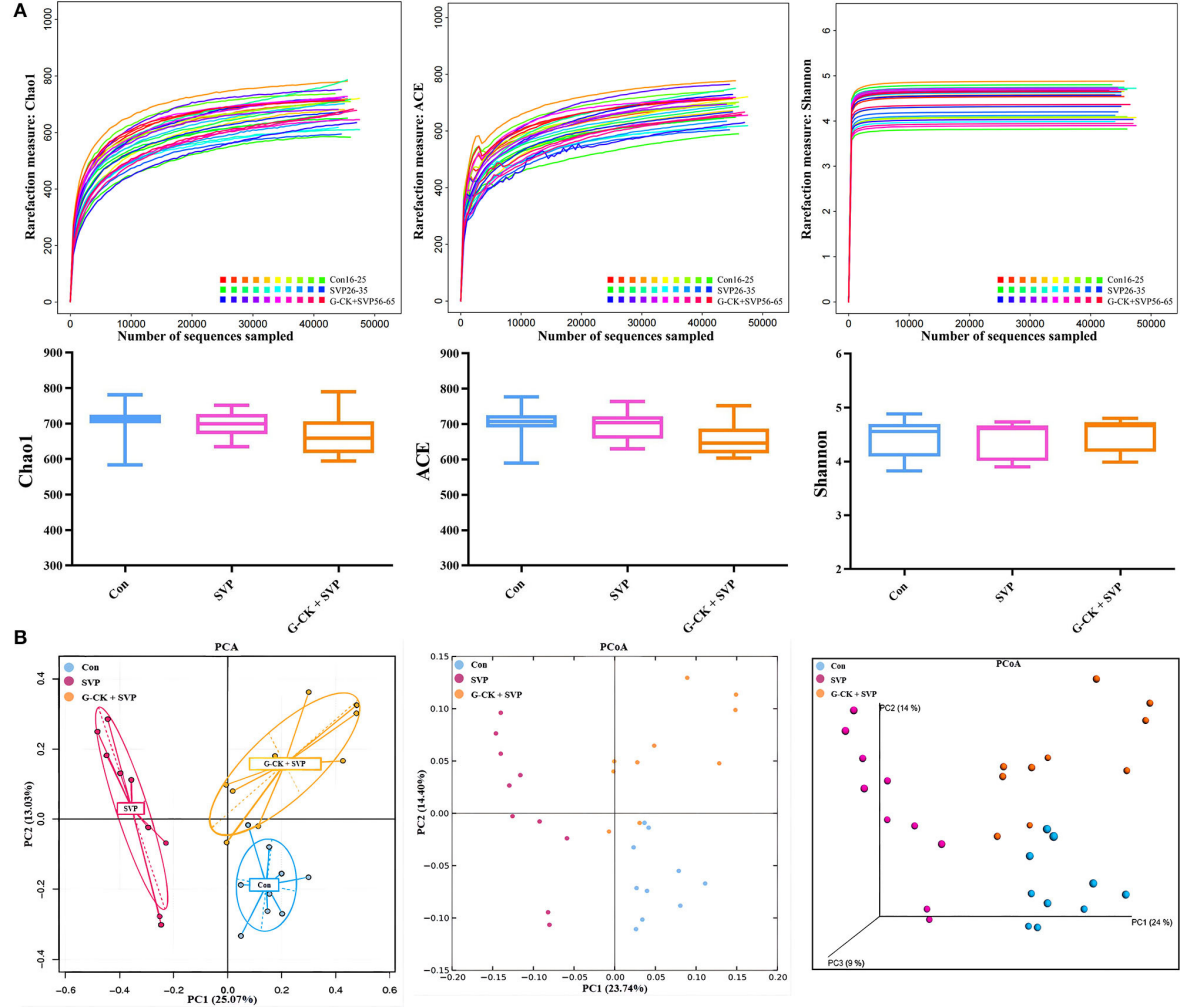
Alpha diversity and beta diversity of gut microbiota. **(A)** Alpha diversity. Data are expressed as median and min-to-max values, *n* = 10. **(B)** Principal components analysis (PCA) and principal coordinate analysis (PCoA), *n* = 10. Con, control; SVP, sodium valproate (500 mg/kg, twice daily); G-CK, ginsenoside compound K (320 mg/kg, once daily).

Alpha-diversity analysis indicated that the values of the Chao1 richness estimator, ACE richness estimator, and the Shannon diversity index showed no significant differences among Con, SVP, and G-CK + SVP groups. The beta diversity (PCA and PCoA) demonstrated clear separation between Con and SVP groups and between SVP and G-CK + SVP groups. These results are presented in Figure 1.

The taxonomic composition of gut bacterial communities in each sample is displayed in Supplementary Figure 1, and the different analyses of relative abundance among groups at the phylum, class, order, family, genus, and species levels are shown in Supplementary Tables 1–6. At the species level, the relative abundance of *Akkermansia muciniphila* (*A. muciniphila*), *Alistipes indistinctus, Bacteroides acidifaciens* (*B. acidifaciens*), *Bacteroides eggerthii, Bifidobacterium pseudolongum* (*B. pseudolongum*), *Lactobacillus reuteri* (*L. reuteri*), and *Prevotella copri* (*P. copri*) were significantly increased in the SVP group (the ratio of SVP vs. Con: 140.749, 8.217, 2.336, 7.806, 248.900, 0.187, and 0.044). Ginsenoside compound K significantly inhibited changes in the relative abundance of *A. muciniphila, B. acidifaciens, L. reuteri*, and *P. copri* caused by SVP (the ratio of G-CK + SVP vs. SVP: 0.007, 0.494, 4.136, and 35.028).

**Figure 2:**
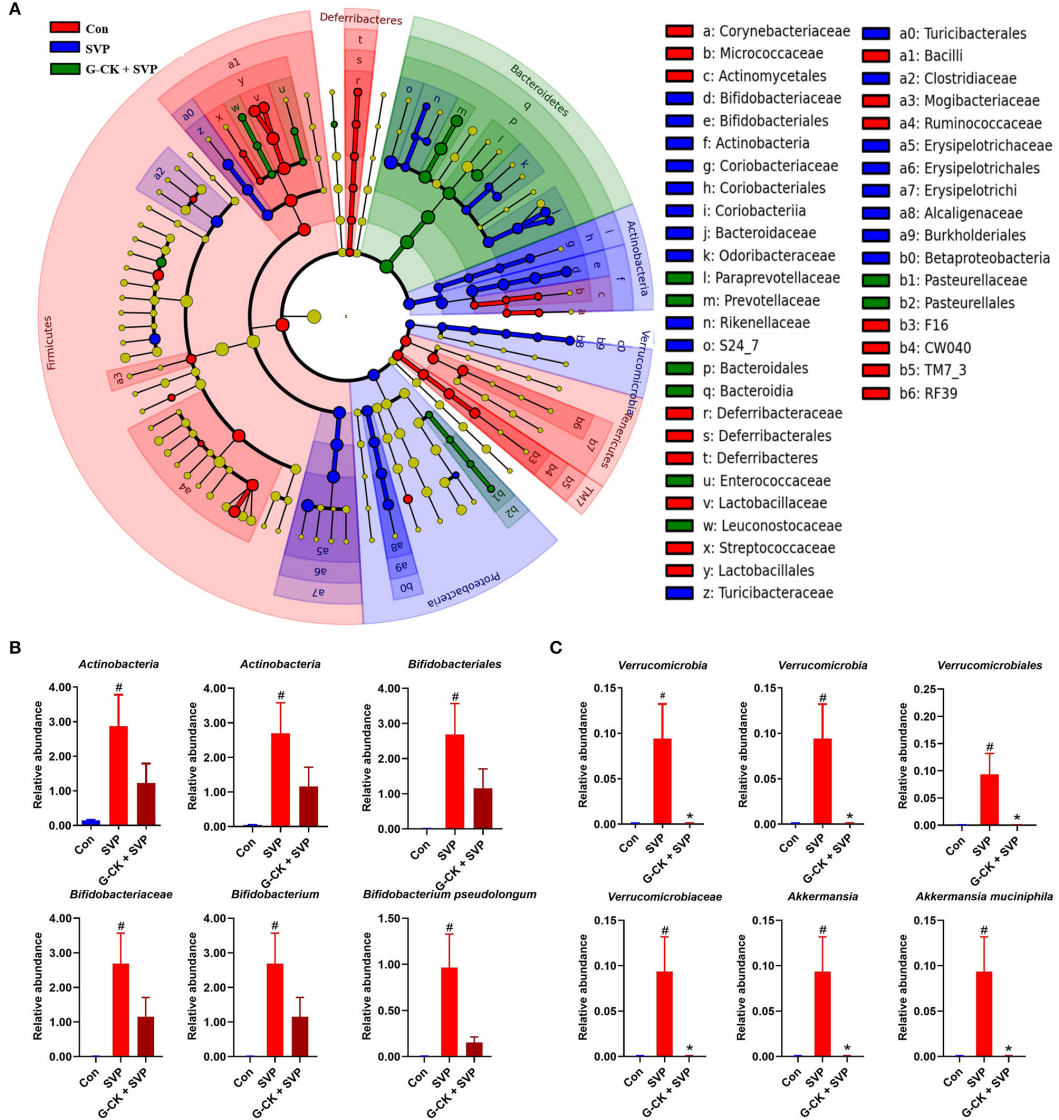
LEfSe analysis of gut microbiota. **(A)** Cladogram representative. The red, blue, and green nodes in the phylogenetic tree represent microbial species that play an important role in the Con, SVP, G-CK + SVP groups, respectively. Yellow nodes represent species with no significant difference. **(B)** The relative abundance of *Actinobacteria, Actinobacteria, Bifidobacteriales, Bifidobacteriaceae, Bifidobacterium*, and *Bifidobacterium pseudolongum*. Values are mean ± SD, *n* = 10. **(C)** The relative abundance of *Verrucomicrobia, Verrucomicrobiae, Verrucomicrobiales, Verrucomicrobiaceae, Akkermansia*, and *Akkermansia muciniphila*. Values are mean ± SD, *n* = 10. Con, control; SVP, sodium valproate (500 mg/kg, twice daily); G-CK, ginsenoside compound K (320 mg/kg, once daily). ^#^*p* < 0.05 vs. Con group, *p < 0.05 vs. SVP group.

The results of LEfSe analysis also showed that the relative abundance of *A. muciniphila* and *B. pseudolongum* were higher in the SVP group than in the Con and G-CK + SVP groups (Figure 2). These results showed that the relative abundance of both *A. muciniphila* and *B. pseudolongum* were correlated with the hepatotoxicity of SVP and the protective effect of G-CK.

### Correlation Between Gut Microbiota Composition and Indices of Liver Function

To investigate the relationship between gut microbiota and liver injury, Spearman's correlation was carried out on the gut microbiota and indices of the liver function of rats from the SVP group. The results of the LEfSe analysis drew our attention to *A. muciniphila* and *B. pseudolongum*. The correlation analysis revealed that *A. muciniphila* was positively correlated with both plasma AST and ALT levels (Table 1). This implicated *A. muciniphila* to be involved in modulating the formation of liver injury induced by SVP.

**Table 1:** Spearman's correlation of gut microbiota and indices of liver function.

**Gut bacteria^a^**	**AST**		**ALT**		**ALP**	
	**r**	***p***	**r**	***p***	**r**	***p***
*Akkermansia muciniphila*	**0.8167**	**0.0108**	**0.7833**	**0.0172**	0.0333	0.9484
*Bifidobacterium pseudolongum*	0.0714	0.8820	0.0857	0.9194	0.1429	0.7825

a *LEfSe analysis showed that the relative abundance of Akkermansia muciniphila and Bifidobacterium pseudolongum were higher in the SVP group than in the Con and G-CK + SVP groups. AST, aspartate transaminase; ALT, alanine aminotransferase; ALP, alkaline phosphatase; Con, control; SVP, sodium valproate (500 mg/kg, twice daily); G-CK, ginsenoside compound K (320 mg/kg, once daily). Bold indicates correlation*.

### Functional Analysis of Gut Microbiota

**Figure 3:**
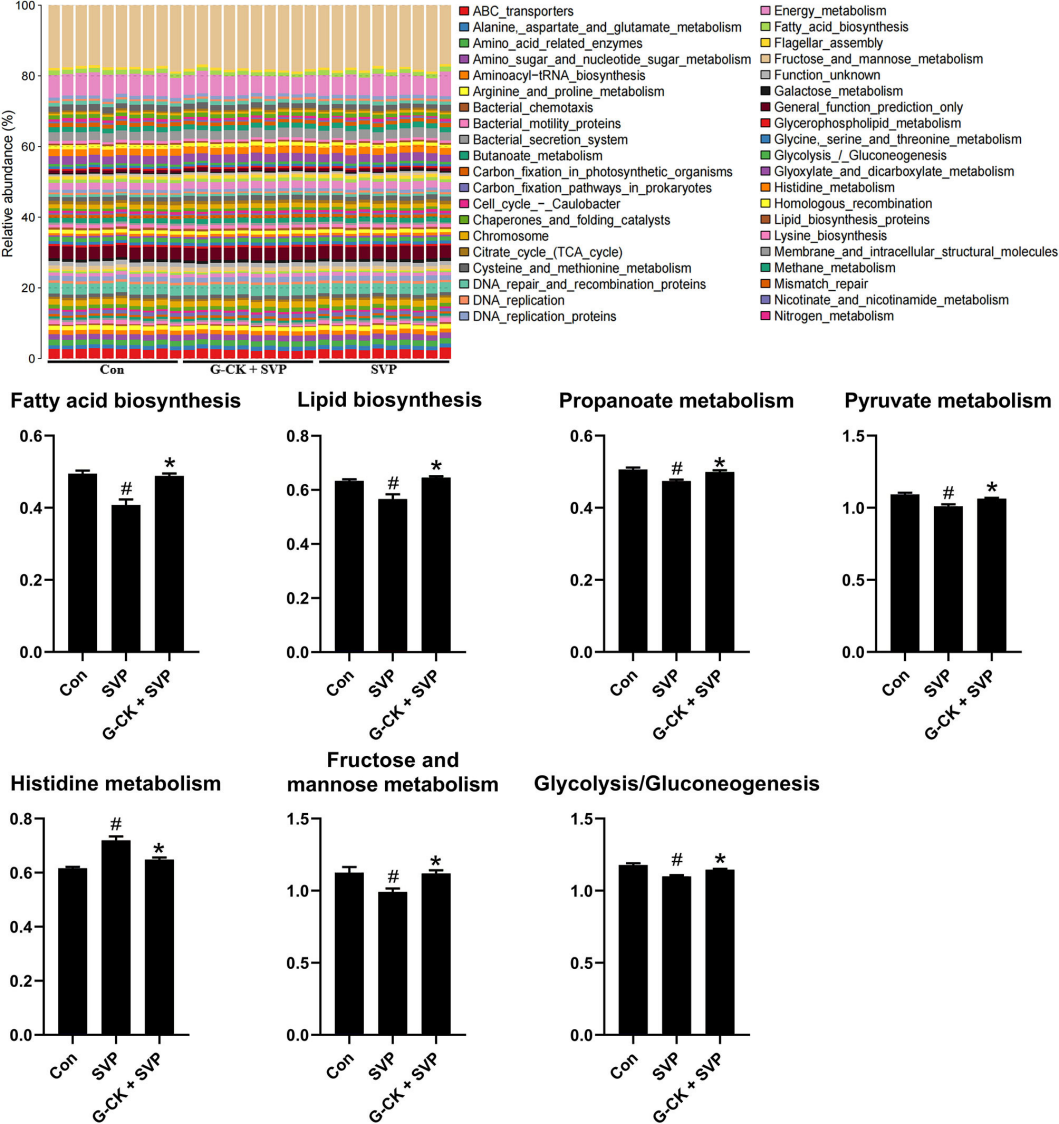
PICRUSt analysis of gut microbiota. Values are mean ± SD, *n* = 10. Con, control; SVP, sodium valproate (500 mg/kg, twice daily); G-CK, ginsenoside compound K (320 mg/kg, once daily). ^#^*p* < 0.05 vs. Con group, *p < 0.05 vs. SVP group.

Analysis by PICRUSt showed that there were seven overlapped differential pathways between SVP vs. Con and G-CK + SVP vs. SVP. These pathways included fatty acid biosynthesis, lipid biosynthesis, propanoate metabolism, histidine metabolism, glycolysis/gluconeogenesis, pyruvate metabolism, and fructose and mannose metabolism (Figure 3). Following this analysis, four pathways including the fatty acid biosynthesis, lipid biosynthesis, glycolysis/gluconeogenesis, and pyruvate metabolism were found to be negatively correlated with both plasma AST and ALT levels in the SVP group (Table 2). In addition, both the glycolysis/gluconeogenesis and pyruvate metabolism pathways were negatively related to the relative abundance of *A. muciniphila* in the SVP group (*r* = −0.7842, *p* = 0.0098; *r* = −0.6687, *p* = 0.0399, respectively). This suggests that the altered levels of glycolysis/gluconeogenesis and pyruvate metabolism likely mediate the role of *A. muciniphila* in SVP-induced hepatotoxicity and the protective effect of G-CK.

**Table 2:** Spearman's correlation of pathways and indices of liver function.

**Pathways^a^**	**AST**		**ALT**		**ALP**	
	**r**	***p***	**r**	***p***	**r**	***p***
Fatty acid biosynthesis	**−0.7000**	**0.0433**	**−0.7500**	**0.0255**	−0.1879	0.6073
Lipid biosynthesis	**−0.7000**	**0.0433**	**−0.7500**	**0.0255**	−0.2606	0.4697
Propanoate metabolism	−0.4167	0.2696	−0.1667	0.6777	−0.0667	0.8651
Histidine metabolism	0.2500	0.5206	0.1333	0.7435	−0.2121	0.5603
Glycolysis/gluconeogenesis	**−0.8667**	**0.0045**	**−0.8333**	**0.0083**	0.0182	0.9730
Pyruvate metabolism	**−0.8167**	**0.0108**	**−0.8000**	**0.0138**	−0.1152	0.7589
Fructose and mannose metabolism	0.2000	0.6134	0.4000	0.2912	0.0424	0.9184

a *Overlapped differential pathways between SVP vs. Con and G-CK + SVP vs. SVP estimated. AST, aspartate transaminase; ALT, alanine aminotransferase; ALP, alkaline phosphatase; Con, control; SVP, sodium valproate (500 mg/kg, twice daily); G-CK, ginsenoside compound K (320 mg/kg, once daily). Bold indicates correlation*.

## Discussion

This study found that SVP caused gut microbiota dysbiosis in the rat, while G-CK could partially ameliorate these changes. At the species level, SVP induced an extremely high accumulation of both *A. muciniphila* and *B. pseudolongum*, and the increased *A. muciniphila* was almost completely reversed by treatment with G-CK. The relative abundance of *A. muciniphila* was strongly positively correlated with AST and ALT levels. PICRUSt analysis showed that there were seven overlapped differential pathways between SVP vs. Con and G-CK + SVP vs. SVP, of which four pathways including fatty acid biosynthesis, lipid biosynthesis, glycolysis/gluconeogenesis, and pyruvate metabolism were found to be negatively correlated with AST and ALT levels. In addition, the glycolysis/gluconeogenesis and pyruvate metabolism were negatively related to the relative abundance of *A. muciniphila*.

*Akkermansia muciniphila*, an intestinal symbiont colonizing in the mucosal layer, can use mucin as its sole carbon, nitrogen, and energy source. This feature renders it to be a promising candidate in probiotic formulations, as it is known to have an important value in metabolic modulation, immune regulation, and protection of gut health (Zhai et al., 2019; Zhang et al., 2019). Although many studies have shown the beneficial effects of *A. muciniphila*, some researchers have taken the opposite view of the species. For instance, alcohol-treated mice have shown higher intestinal levels of *A. muciniphila* (Yan et al., 2011). Additionally, in both specific-pathogen-free and germ-free *IL10*^−/−^ mice, *A. muciniphila* administration was sufficient for promoting intestinal inflammation (Seregin et al., 2017). A diet including heme has also been shown to lead to a damaged gut epithelium, compensatory hyperproliferation, and an 8-fold increase in *A. muciniphila*. Broad-spectrum antibiotics were able to reduce the abundance of *A. muciniphila* more than 1,000-fold, and this was accompanied by eliminating the observed hyperproliferation phenotype (Ijssennagger et al., 2015). High L-carnitine supplementation has also been shown to induce the accumulation of *A. muciniphila*, and the relative abundance of *A. muciniphila* was strongly positively correlated with AST, IL-1, TNF-α, TNF-β, and MDA levels in male mice (Wu et al., 2020). In this study, we observed that the repeated oral gavage of SVP induced a 140-fold elevation in the relative abundance of *A. muciniphila*, which was almost completely reversed by the administration of G-CK. The relative abundance of *A. muciniphila* was strongly positively correlated with AST and ALT levels. This suggests that decreasing the population level of *A. muciniphila* might mediate the protective effects of G-CK against the hepatotoxicity of SVP, and thus researchers should give further recognition to the potentially adverse effects of *A. muciniphila*.

Furthermore, the functional pathways of gut microbiota were disturbed by SVP exposures. Seven of the changed pathways caused by SVP, including fatty acid biosynthesis, lipid biosynthesis, propanoate metabolism, histidine metabolism, glycolysis/gluconeogenesis, pyruvate metabolism, and fructose and mannose metabolism, were significantly inhibited by G-CK. Meanwhile, the fatty acid biosynthesis, lipid biosynthesis, glycolysis/gluconeogenesis, and pyruvate metabolism were negatively correlated with both AST and ALT levels. The glycolysis/gluconeogenesis and pyruvate metabolism were negatively correlated with the relative abundance of *A. muciniphila*. Pyruvate metabolism is known to be closely related to glycolysis/gluconeogenesis with pyruvate as the main connecting node. It was reported that both valproic acid and SVP could increase the liver tissue and blood concentrations of pyruvate and strongly inhibit its oxidation (Daniels et al., 2004; Huo et al., 2014; Kudin et al., 2017). Pyruvate oxidation defects are among the most frequent causes of deficiencies in mitochondrial energy metabolism, thus an important feature of classical mitochondrial diseases (Sperl et al., 2015). Mitochondrial dysfunction is a well-known mechanism of SVP-induced hepatotoxicity (Ramachandran et al., 2018). Based on this, we speculate that the decrease in pyruvate metabolism mediated by the increase in *A. muciniphila* might be involved in the hepatotoxicity of SVP. Unfortunately, we did not measure the concentrations of pyruvate in plasma and fecal samples due to sample insufficiency. We have designed experiments to further study the role of *A. muciniphila* in the hepatotoxicity of SVP, which has been supported by the China Postdoctoral Science Foundation. This will provide new insights into the mechanisms, early warning signs, and possible prevention strategies for SVP-induced hepatotoxicity.

In conclusion, SVP-induced hepatotoxicity was related to gut microbiota dysbiosis, namely, the increased relative abundance of *A. muciniphila* and decreased glycolysis/gluconeogenesis and pyruvate metabolism pathways. Ginsenoside compound K could significantly improve the outcomes caused by SVP exposure. The results in this study suggest that the altered levels of *A. muciniphila* resulted in changes in glycolysis/gluconeogenesis and pyruvate metabolism, and this may modulate the hepatotoxicity of SVP and the protective effect of G-CK administration against SVP-induced hepatotoxicity.

## Data Availability Statement

The datasets presented in this study can be found in online repositories. The names of the repository/repositories and accession number(s) can be found below: NCBI—PRJNA837380.

## Ethics Statement

The animal study was reviewed and approved by Laboratory Animal Ethics Committee of Servicebio.

## Author Contributions

LZ, LC, and DO designed the study. LZ performed the experiments, analyzed the data, and drafted the manuscript. LZ, XZ, JL, LC, and DO revised the manuscript. All authors read and approved the final manuscript.

## Funding

This study was supported by the China Postdoctoral Science Foundation (No. 2021M702910), the Key R&D Programs of Hunan Province (No. 2019SK2241), the Innovation and Entrepreneurship Investment Project in Hunan Province (No. 2019GK5020), the International Scientific and Technological Innovation Cooperation Base for Bioanalysis of Complex Matrix Samples in Hunan Province (No. 2019CB1014), and the Science and Technology Project of Changsha (No. kh1902002).

## Conflict of Interest

The authors declare that the research was conducted in the absence of any commercial or financial relationships that could be construed as a potential conflict of interest.

## Publisher's Note

All claims expressed in this article are solely those of the authors and do not necessarily represent those of their affiliated organizations, or those of the publisher, the editors and the reviewers. Any product that may be evaluated in this article, or claim that may be made by its manufacturer, is not guaranteed or endorsed by the publisher.

## Supplementary Material

Supplementary Figure 1 Relative abundance of gut microbiota in each sample at phylum, class, order, family, genus, and species levels, *n* = 10 in each group. Con, control; SVP, sodium valproate (500 mg/kg, twice daily); G-CK, ginsenoside compound K (320 mg/kg, once daily).

Supplementary Table 1 Statistics values for relative abundance of bacterial phylum.

Supplementary Table 2 Statistics values for relative abundance of bacterial class.

Supplementary Table 3 Statistics values for relative abundance of bacterial order.

Supplementary Table 4 Statistics values for relative abundance of bacterial family.

Supplementary Table 5 Statistics values for relative abundance of bacterial genus.

Supplementary Table 6 Statistics values for relative abundance of bacterial species.
