# Supplementary figures and images for: Gut Microbiota Modulates the Protective Role of Ginsenoside Compound K Against Sodium Valproate-Induced Hepatotoxicity in Rat
Source: Front Microbiol. 2022 Jul 7;13:936585. doi: 10.3389/fmicb.2022.936585 (PMC9302921; doi:10.3389/fmicb.2022.936585)

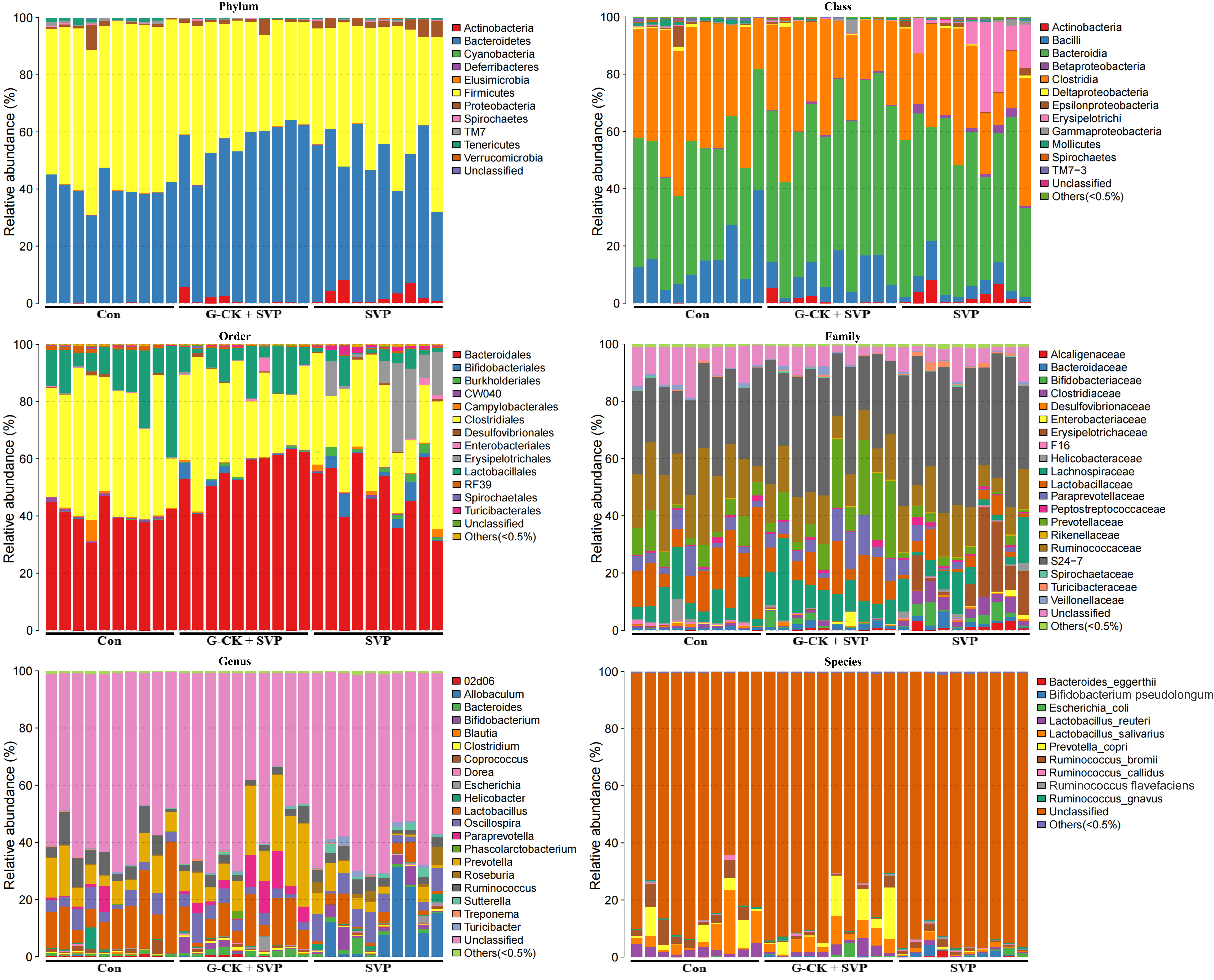

Supplement: Supplementary Figure 1 — Relative abundance of gut microbiota in each sample at phylum, class, order, family, genus, and species levels, n = 10 in each group. Con, control; SVP, sodium valproate (500 mg/kg, twice daily); G-CK, ginsenoside compound K (320 mg/kg, once daily). [file Image_1.JPEG]
