# Supplementary material for: Gut Microbiota Modulates the Protective Role of Ginsenoside Compound K Against Sodium Valproate-Induced Hepatotoxicity in Rat
Source: Front Microbiol. 2022 Jul 7;13:936585. doi: 10.3389/fmicb.2022.936585 (PMC9302921; doi:10.3389/fmicb.2022.936585)
Supplement: Supplementary Table 1 — Statistics values for relative abundance of bacterial phylum. [file Table_1.DOCX]

Supplementary Table 1. Statistics values for relative abundance of bacterial phylum

| Phylum | SVP *vs.* Con | | |  | HCK + SVP *vs.* SVP | | |
| --- | --- | --- | --- | --- | --- | --- | --- |
|  | Ratio | *p* | FDR |  | Ratio | *p* | FDR |
| *Actinobacteria* | 19.664 | <0.001 | <0.001^#^ |  | 0.426 | 0.035 | 0.085 |
| *Bacteroidetes* | 1.216 | 0.052 | 0.090 |  | 1.150 | 0.143 | 0.245 |
| *Cyanobacteria* | 0.789 | 0.105 | 0.134 |  | 1.174 | 0.970 | 0.971 |
| *Deferribacteres* | 0.317 | 0.732 | 0.798 |  | 0.057 | 0.029 | 0.085 |
| *Elusimicrobia* | 0.959 | 0.393 | 0.472 |  | 1.095 | 0.315 | 0.472 |
| *Firmicutes* | 0.792 | 0.011 | 0.034^#^ |  | 0.913 | 0.393 | 0.524 |
| ***Proteobacteria*** | **1.851** | **0.011** | **0.034^#^** |  | **0.498** | **0.009** | **0.036^*^** |
| *Spirochaetes* | 0.676 | 1.000 | 1.000 |  | 1.220 | 0.791 | 0.949 |
| *Tenericutes* | 0.322 | 0.015 | 0.035^#^ |  | 1.026 | 0.063 | 0.971 |
| *TM7* | 0.440 | 0.043 | 0.087 |  | 0.340 | 0.971 | 0.126 |
| ***Verrucomicrobia*** | **141.761** | **0.008** | **0.034^#^** |  | **0.007** | **0.008** | **0.036^*^** |

Con, control; SVP, sodium valproate (500 mg/kg, twice daily); G-CK, ginsenoside compound K (320 mg/kg, once daily). ^#^ FDR <0.05 *vs.* Con group, ^*^ FDR <0.05 *vs.* SVP group.
