# Supplementary material for: Gut Microbiota Modulates the Protective Role of Ginsenoside Compound K Against Sodium Valproate-Induced Hepatotoxicity in Rat
Source: Front Microbiol. 2022 Jul 7;13:936585. doi: 10.3389/fmicb.2022.936585 (PMC9302921; doi:10.3389/fmicb.2022.936585)
Supplement: Supplementary Table 2 — Statistics values for relative abundance of bacterial class. [file Table_2.DOCX]

Supplementary Table 2. Statistics values for relative abundance of bacterial class

| Class | SVP *vs.* Con | | |  | HCK + SVP *vs.* SVP | | |
| --- | --- | --- | --- | --- | --- | --- | --- |
|  | Ratio | *p* | FDR |  | Ratio | *p* | FDR |
| *Actinobacteria* | 57.748 | <0.001 | <0.001^#^ |  | 0.429 | 0.035 | 0.124 |
| *Alphaproteobacteria* | 1.729 | 0.225 | 0.337 |  | 0.280 | 0.849 | 1.000 |
| *Bacilli* | 0.300 | 0.001 | 0.011^#^ |  | 2.071 | 0.052 | 0.157 |
| *Bacteroidia* | 1.215 | 0.052 | 0.122 |  | 1.149 | 0.165 | 0.353 |
| *Betaproteobacteria* | 9.570 | 0.052 | 0.122 |  | 0.299 | 0.190 | 0.363 |
| *Clostridia* | 0.732 | 0.052 | 0.122 |  | 1.040 | 0.971 | 1.000 |
| *Coriobacteriia* | 1.741 | 0.143 | 0.250 |  | 0.378 | 0.003 | 0.030^*^ |
| *Deltaproteobacteria* | 0.733 | 0.436 | 0.538 |  | 0.846 | 1.000 | 1.000 |
| *Elusimicrobia* | 0.959 | 0.393 | 0.516 |  | 1.095 | 0.315 | 0.551 |
| *Epsilonproteobacteria* | 0.867 | 0.481 | 0.561 |  | 0.204 | 0.029 | 0.123 |
| ***Erysipelotrichi*** | **51.114** | **0.003** | **0.020^#^** |  | **0.013** | **0.001** | **0.015^*^** |
| *Gammaproteobacteria* | 12.195 | 0.257 | 0.359 |  | 1.379 | 0.579 | 0.868 |
| *Mollicutes* | 0.322 | 0.015 | 0.062 |  | 1.026 | 0.971 | 1.000 |
| *RF3* | 1.200 | 0.825 | 0.866 |  | 1.203 | 0.966 | 1.000 |
| *Spirochaetes* | 0.676 | 1.000 | 1.000 |  | 1.22 | 0.791 | 1.000 |
| *TM7_3* | 0.440 | 0.043 | 0.122 |  | 0.340 | 0.063 | 0.165 |
| *Verrucomicrobiae* | 140.749 | 0.010 | 0.053 |  | 0.007 | 0.013 | 0.088 |
| *4C0d_2* | 0.789 | 0.105 | 0.201 |  | 1.174 | 0.970 | 1.000 |

Con, control; SVP, sodium valproate (500 mg/kg, twice daily); G-CK, ginsenoside compound K (320 mg/kg, once daily). ^#^ FDR <0.05 *vs.* Con group, ^*^ FDR <0.05 *vs.* SVP group.
