# Supplementary material for: Gut Microbiota Modulates the Protective Role of Ginsenoside Compound K Against Sodium Valproate-Induced Hepatotoxicity in Rat
Source: Front Microbiol. 2022 Jul 7;13:936585. doi: 10.3389/fmicb.2022.936585 (PMC9302921; doi:10.3389/fmicb.2022.936585)
Supplement: Supplementary Table 3 — Statistics values for relative abundance of bacterial order. [file Table_3.DOCX]

Supplementary Table 3. Statistics values for relative abundance of bacterial order

| Order | SVP *vs.* Con | | |  | HCK + SVP *vs.* SVP | | |
| --- | --- | --- | --- | --- | --- | --- | --- |
|  | Ratio | *p* | FDR |  | Ratio | *p* | FDR |
| *Actinomycetales* | 0.261 | <0.001 | 0.003^#^ |  | 0.744 | 0.436 | 0.667 |
| *Anaeroplasmatales* | 1.449 | 0.756 | 0.855 |  | 4.421 | 0.211 | 0.365 |
| *Bacillales* | 0.923 | 0.909 | 0.945 |  | 0.503 | 0.180 | 0.353 |
| *Bacteroidales* | 1.215 | 0.052 | 0.124 |  | 1.149 | 0.165 | 0.353 |
| *Bifidobacteriales* | 296.570 | <0.001 | <0.001^#^ |  | 0.428 | 0.035 | 0.102 |
| *Burkholderiales* | 9.570 | 0.052 | 0.124 |  | 0.299 | 0.190 | 0.353 |
| *CW040* | 0.440 | 0.043 | 0.124 |  | 0.340 | 0.063 | 0.164 |
| *Campylobacterales* | 0.867 | 0.481 | 0.626 |  | 0.204 | 0.029 | 0.095 |
| *Clostridiales* | 0.732 | 0.052 | 0.124 |  | 1.040 | 0.971 | 1.000 |
| *Coriobacteriales* | 1.741 | 0.143 | 0.266 |  | 0.378 | 0.003 | 0.019^*^ |
| *Deferribacterales* | 0.317 | 0.732 | 0.855 |  | 0.057 | 0.029 | 0.095 |
| *Desulfovibrionales* | 0.733 | 0.436 | 0.596 |  | 0.846 | 1.000 | 1.000 |
| *Elusimicrobiales* | 0.959 | 0.393 | 0.568 |  | 1.095 | 0.315 | 0.512 |
| *Enterobacteriales* | 12.404 | 0.257 | 0.393 |  | 1.362 | 0.684 | 0.936 |
| ***Erysipelotrichales*** | **51.114** | **0.003** | **0.015^#^** |  | **0.013** | **0.001** | **0.012^*^** |
| *Lactobacillales* | 0.226 | <0.001 | 0.003^#^ |  | 2.737 | 0.015 | 0.064 |
| *ML615J_28* | 1.200 | 0.825 | 0.893 |  | 1.203 | 0.966 | 1.000 |
| *Pasteurellales* | 0.334 | 0.584 | 0.723 |  | 36.751 | 0.001 | 0.012^*^ |
| *RF32* | 1.729 | 0.225 | 0.365 |  | 0.280 | 0.849 | 1.000 |
| *RF39* | 0.318 | 0.015 | 0.055 |  | 1.002 | 0.853 | 1.000 |
| *Spirochaetales* | 0.676 | 1.000 | 1.000 |  | 1.220 | 0.791 | 1.000 |
| ***Turicibacterales*** | **5.353** | **<0.001** | **0.003^#^** |  | **0.146** | **0.001** | **0.012^*^** |
| *Verrucomicrobiales* | 140.749 | 0.010 | 0.043^#^ |  | 0.007 | 0.013 | 0.064 |
| *YS2* | 0.789 | 0.105 | 0.210 |  | 1.174 | 0.970 | 1.000 |

Con, control; SVP, sodium valproate (500 mg/kg, twice daily); G-CK, ginsenoside compound K (320 mg/kg, once daily). ^#^ FDR <0.05 *vs.* Con group, ^*^ FDR <0.05 *vs.* SVP group.
