# Supplementary material for: Gut Microbiota Modulates the Protective Role of Ginsenoside Compound K Against Sodium Valproate-Induced Hepatotoxicity in Rat
Source: Front Microbiol. 2022 Jul 7;13:936585. doi: 10.3389/fmicb.2022.936585 (PMC9302921; doi:10.3389/fmicb.2022.936585)
Supplement: Supplementary Table 4 — Statistics values for relative abundance of bacterial family. [file Table_4.DOCX]

Supplementary Table 4. Statistics values for relative abundance of bacterial family

| Family | SVP *vs.* Con | | |  | HCK + SVP *vs.* SVP | | |
| --- | --- | --- | --- | --- | --- | --- | --- |
|  | Ratio | *p* | FDR |  | Ratio | *p* | FDR |
| *Alcaligenaceae* | 17.405 | 0.015 | 0.036^#^ |  | 0.298 | 0.190 | 0.325 |
| *Anaeroplasmataceae* | 1.449 | 0.756 | 0.882 |  | 4.421 | 0.211 | 0.345 |
| *Bacteroidaceae* | 2.007 | 0.089 | 0.166 |  | 0.958 | 0.529 | 0.657 |
| *Bifidobacteriaceae* | 296.570 | <0.001 | <0.001^#^ |  | 0.428 | 0.035 | 0.081 |
| *Christensenellaceae* | 0.943 | 0.912 | 0.935 |  | 0.750 | 0.280 | 0.410 |
| ***Clostridiaceae*** | **6.200** | **<0.001** | **<0.001^#^** |  | **0.129** | **<0.001** | **0.002^*^** |
| *Coriobacteriaceae* | 1.741 | 0.143 | 0.255 |  | 0.378 | 0.003 | 0.015^*^ |
| *Deferribacteraceae* | 0.310 | 0.732 | 0.882 |  | 0.057 | 0.029 | 0.070 |
| *Dehalobacteriaceae* | 0.903 | 0.853 | 0.921 |  | 0.899 | 0.739 | 0.866 |
| *Desulfovibrionaceae* | 0.733 | 0.436 | 0.596 |  | 0.846 | 1.000 | 1.000 |
| *Elusimicrobiaceae* | 0.959 | 0.393 | 0.556 |  | 1.095 | 0.315 | 0.445 |
| *Enterobacteriaceae* | 12.404 | 0.257 | 0.405 |  | 1.362 | 0.684 | 0.825 |
| ***Erysipelotrichaceae*** | **51.114** | **0.003** | **0.013^#^** |  | **0.013** | **0.001** | **0.008^*^** |
| *F16* | 0.440 | 0.043 | 0.089 |  | 0.340 | 0.063 | 0.129 |
| *Helicobacteraceae* | 0.867 | 0.481 | 0.617 |  | 0.204 | 0.029 | 0.070 |
| *Lachnospiraceae* | 0.803 | 0.481 | 0.617 |  | 1.653 | 0.143 | 0.279 |
| *Lactobacillaceae* | 0.226 | <0.001 | 0.003^#^ |  | 2.727 | 0.019 | 0.058 |
| *Micrococcaceae* | 0.193 | 0.003 | 0.013^#^ |  | 2.168 | 0.021 | 0.060 |
| *Mogibacteriaceae* | 0.549 | 0.019 | 0.042^#^ |  | 1.196 | 0.481 | 0.617 |
| *Odoribacteraceae* | 5.709 | <0.001 | <0.001^#^ |  | 0.665 | 0.247 | 0.390 |
| *Paraprevotellaceae* | 0.460 | 0.063 | 0.123 |  | 3.796 | 0.005 | 0.024^*^ |
| *Pasteurellaceae* | 0.334 | 0.584 | 0.726 |  | 36.751 | 0.001 | 0.010^*^ |
| *Peptococcaceae* | 0.938 | 0.796 | 0.882 |  | 0.699 | 0.280 | 0.410 |
| *Peptostreptococcaceae* | 1.083 | 0.796 | 0.882 |  | 0.778 | 0.393 | 0.520 |
| *Porphyromonadaceae* | 1.024 | 0.280 | 0.425 |  | 0.826 | 1.000 | 1.000 |
| ***Prevotellaceae*** | **0.327** | **0.009** | **0.029^#^** |  | **6.723** | **<0.001** | **0.007^*^** |
| *Rikenellaceae* | 2.995 | 0.001 | 0.007^#^ |  | 0.713 | 0.015 | 0.051 |
| *Ruminococcaceae* | 0.610 | 0.005 | 0.021^#^ |  | 1.048 | 0.853 | 0.921 |
| *S24_7* | 1.478 | 0.002 | 0.009^#^ |  | 0.783 | 0.023 | 0.063 |
| *Spirochaetaceae* | 0.676 | 1.000 | 1.000 |  | 1.220 | 0.791 | 0.901 |
| *Staphylococcaceae* | 0.923 | 0.909 | 0.935 |  | 0.503 | 0.180 | 0.321 |
| *Streptococcaceae* | 0.342 | 0.009 | 0.029^#^ |  | 1.712 | 0.063 | 0.129 |
| ***Turicibacteraceae*** | **5.353** | **<0.001** | **0.003^#^** |  | **0.146** | **0.001** | **0.008^*^** |
| *Veillonellaceae* | 0.413 | 0.029 | 0.062 |  | 2.082 | 0.353 | 0.482 |
| *Verrucomicrobiaceae* | 140.749 | 0.010 | 0.029^#^ |  | 0.007 | 0.013 | 0.051 |

Con, control; SVP, sodium valproate (500 mg/kg, twice daily); G-CK, ginsenoside compound K (320 mg/kg, once daily). ^#^ FDR <0.05 *vs.* Con group, ^*^ FDR <0.05 *vs.* SVP group.
