# Supplementary material for: Gut Microbiota Modulates the Protective Role of Ginsenoside Compound K Against Sodium Valproate-Induced Hepatotoxicity in Rat
Source: Front Microbiol. 2022 Jul 7;13:936585. doi: 10.3389/fmicb.2022.936585 (PMC9302921; doi:10.3389/fmicb.2022.936585)
Supplement: Supplementary Table 5 — Statistics values for relative abundance of bacterial genus. [file Table_5.DOCX]

Supplementary Table 5. Statistics values for relative abundance of bacterial genus

| Genus | SVP *vs.* Con | | |  | HCK + SVP *vs.* SVP | | |
| --- | --- | --- | --- | --- | --- | --- | --- |
|  | Ratio | *p* | FDR |  | Ratio | *p* | FDR |
| *AF12* | 11.485 | 0.036 | 0.099 |  | 0.024 | 0.005 | 0.023^*^ |
| *Adlercreutzia* | 1.282 | 0.063 | 0.149 |  | 0.357 | <0.001 | 0.001^*^ |
| *Aggregatibacter* | 0.334 | 0.584 | 0.779 |  | 36.751 | 0.001 | 0.011^*^ |
| ***Akkermansia*** | **140.749** | **0.010** | **0.037^#^** |  | **0.007** | **0.013** | **0.049^*^** |
| *Alistipes* | 8.217 | 0.007 | 0.030^#^ |  | 0.545 | 0.304 | 0.494 |
| ***Allobaculum*** | **226.629** | **<0.001** | **0.001^#^** |  | **0.007** | **0.001** | **0.008^*^** |
| *Anaeroplasma* | 1.449 | 0.756 | 0.819 |  | 4.428 | 0.211 | 0.391 |
| *Anaerostipes* | 1.415 | 0.247 | 0.429 |  | 1.919 | 0.970 | 0.990 |
| *Bacteroides* | 2.007 | 0.089 | 0.193 |  | 0.964 | 0.529 | 0.671 |
| *Bifidobacterium* | 296.570 | <0.001 | 0.001^#^ |  | 0.428 | 0.035 | 0.088 |
| *Blautia* | 0.128 | 0.007 | 0.030^#^ |  | 4.957 | 0.199 | 0.382 |
| *Butyricicoccus* | 1.473 | 0.739 | 0.818 |  | 1.515 | 0.247 | 0.444 |
| *Butyricimonas* | 3.946 | 0.001 | 0.006^#^ |  | 0.939 | 0.739 | 0.854 |
| *Christensenella* | 0.500 | 0.428 | 0.602 |  | 1.376 | 0.912 | 0.968 |
| *Clostridium* | 1.000 | 0.853 | 0.888 |  | 0.270 | 0.399 | 0.561 |
| *Coprobacillus* | 0.442 | 0.219 | 0.393 |  | 2.065 | 0.075 | 0.169 |
| *Coprococcus* | 0.357 | 0.052 | 0.130 |  | 0.099 | 0.001 | 0.008^*^ |
| *Corynebacterium* | 0.310 | 0.015 | 0.046^#^ |  | 2.007 | 0.273 | 0.469 |
| *Defluviitalea* | 0.754 | 0.631 | 0.792 |  | 0.897 | 0.571 | 0.690 |
| *Dehalobacterium* | 1.017 | 0.853 | 0.888 |  | 1.092 | 0.847 | 0.937 |

Con, control; SVP, sodium valproate (500 mg/kg, twice daily); G-CK, ginsenoside compound K (320 mg/kg, once daily). ^#^ FDR <0.05 *vs.* Con group, ^*^ FDR <0.05 *vs.* SVP group.
