# Supplementary material for: Gut Microbiota Modulates the Protective Role of Ginsenoside Compound K Against Sodium Valproate-Induced Hepatotoxicity in Rat
Source: Front Microbiol. 2022 Jul 7;13:936585. doi: 10.3389/fmicb.2022.936585 (PMC9302921; doi:10.3389/fmicb.2022.936585)
Supplement: Supplementary Table 6 — Statistics values for relative abundance of bacterial species. [file Table_6.DOCX]

Supplementary Table 6. Statistics values for relative abundance of bacterial species

| Species | SVP *vs.* Con | | |  | HCK + SVP *vs.* SVP | | |
| --- | --- | --- | --- | --- | --- | --- | --- |
|  | Ratio | *p* | FDR |  | Ratio | *p* | FDR |
| *Aggregatibacter pneumotropica* | 0.334 | 0.584 | 0.911 |  | 36.751 | 0.001 | 0.020^*^ |
| ***Akkermansia muciniphila*** | **140.749** | **0.010** | **0.033^#^** |  | **0.007** | **0.013** | **0.037^*^** |
| *Alistipes indistinctus* | 8.217 | 0.007 | 0.025^#^ |  | 0.545 | 0.304 | 0.439 |
| ***Bacteroides acidifaciens*** | **2.336** | **0.002** | **0.011^#^** |  | **0.494** | **0.004** | **0.034^*^** |
| *Bacteroides eggerthii* | 7.806 | 0.001 | 0.007^#^ |  | 0.246 | 0.247 | 0.428 |
| *Bacteroides uniformis* | 1.418 | 0.971 | 0.971 |  | 1.670 | 0.247 | 0.428 |
| *Bifidobacterium pseudolongum* | 248.900 | <0.001 | 0.002^#^ |  | 0.159 | 0.011 | 0.054 |
| *Butyricicoccus pullicaecorum* | 1.473 | 0.739 | 0.915 |  | 1.515 | 0.247 | 0.428 |
| *Clostridium colinum* | 0.488 | 0.670 | 0.915 |  | 0.498 | 0.584 | 0.723 |
| *Clostridium hungatei* | 1.087 | 0.879 | 0.971 |  | 0.765 | 0.675 | 0.798 |
| *Clostridium methylpentosum* | 0.623 | 0.105 | 0.273 |  | 0.910 | 0.853 | 0.888 |
| *Clostridium ruminantium* | 0.224 | 0.244 | 0.488 |  | 4.569 | 0.035 | 0.113 |
| *Defluviitalea saccharophila* | 0.754 | 0.631 | 0.911 |  | 2.007 | 0.273 | 0.428 |
| *Escherichia coli* | 13.229 | 0.206 | 0.447 |  | 1.373 | 0.427 | 0.585 |
| ***Lactobacillus reuteri*** | **0.187** | **<0.001** | **0.002^#^** |  | **4.136** | **0.005** | **0.034^*^** |
| *Mucispirillum schaedleri* | 0.317 | 0.732 | 0.915 |  | 0.057 | 0.029 | 0.108 |
| *Parabacteroides distasonis* | 1.400 | 0.796 | 0.941 |  | 0.819 | 0.912 | 0.912 |
| ***Prevotella copri*** | **0.044** | **0.001** | **0.007^#^** |  | **35.028** | **0.002** | **0.020^*^** |
| *Ruminococcus albus* | 0.307 | 0.968 | 0.971 |  | 1.343 | 0.511 | 0.665 |
| *Ruminococcus bromii* | 0.306 | 0.029 | 0.083 |  | 0.894 | 0.739 | 0.836 |
| *Ruminococcus callidus* | 1.089 | 0.165 | 0.391 |  | 0.558 | 0.088 | 0.230 |
| *Ruminococcus flavefaciens* | 1.331 | 0.436 | 0.756 |  | 0.601 | 0.280 | 0.428 |
| *Ruminococcus gnavus* | 1.172 | 0.631 | 0.911 |  | 1.167 | 0.853 | 0.888 |
| *Staphylococcus sciuri* | 0.923 | 0.909 | 0.971 |  | 0.503 | 0.180 | 0.390 |

Con, control; SVP, sodium valproate (500 mg/kg, twice daily); G-CK, ginsenoside compound K (320 mg/kg, once daily). ^#^ FDR <0.05 *vs.* Con group, ^*^ FDR <0.05 *vs.* SVP group.
